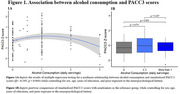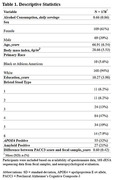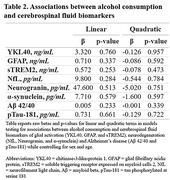# Investigating the role of gut microbiome in alcohol consumption‐associated alterations to cognitive function and biomarkers of Alzheimer’s disease and related dementias

**DOI:** 10.1002/alz.089996

**Published:** 2025-01-09

**Authors:** Darby Peter, Margo B. Heston, Erin M. Jonaitis, Nathaniel A. Chin, Sterling C. Johnson, Sanjay Asthana, Cynthia M. Carlsson, Rob Knight, Rima Kaddurah‐Daouk, Kaj Blennow, Henrik Zetterberg, Federico E. Rey, Barbara B. Bendlin

**Affiliations:** ^1^ Wisconsin Alzheimer's Disease Research Center, University of Wisconsin School of Medicine and Public Health, Madison, WI USA; ^2^ Department of Medicine, University of Wisconsin School of Medicine and Public Health, Madison, WI USA; ^3^ Neuroscience Training Program, University of Wisconsin‐Madison, School of Medicine and Public Health, Madison, WI USA; ^4^ Alzheimer's Disease Research Center, University of Wisconsin‐Madison, Madison, WI USA; ^5^ Wisconsin Alzheimer's Disease Research Center, Madison, WI USA; ^6^ University of Wisconsin School of Medicine and Public Health, Madison, WI USA; ^7^ Center for Microbiome Innovation, University of California San Diego, La Jolla, CA USA; ^8^ Duke University Medical Center, Durham, NC USA; ^9^ Institute of Neuroscience and Physiology, Department of Psychiatry and Neurochemistry, The Sahlgrenska Academy, University of Gothenburg, Mölndal Sweden; ^10^ Institute of Neuroscience and Physiology, Department of Psychiatry and Neurochemistry, The Sahlgrenska Academy, University of Gothenburg, Mölndal, Gothenburg Sweden; ^11^ Department of Bacteriology, University of Wisconsin‐Madison, Madison, WI USA; ^12^ Neuroscience Training Program, University of Wisconsin‐Madison, Madison, WI USA

## Abstract

**Background:**

Heavy alcohol consumption is associated with increased risk for Alzheimer’s disease and related dementias (ADRD), with mixed evidence suggesting a dose‐dependent nonlinear effect of alcohol on ADRD. Potential mechanisms by which alcohol may promote or attenuate brain pathology need further exploration. Although chronic alcohol consumption associates with gut microbiome alterations, it remains unclear whether microbial alterations mediate alcohol‐associated neurodegeneration and cognitive decline. Here we investigated whether the gut microbiome mediates alcohol consumption effects on cognitive function and biomarkers of neurodegeneration, glial activation, and AD.

**Method:**

Cognitively unimpaired adults (n=178, Wisconsin Registry for Alzheimer’s Prevention and Wisconsin Alzheimer’s Disease Research Center; Table 1) underwent neuropsychological evaluation, provided stool samples, and reported average daily alcohol consumption via questionnaire; a subset also underwent lumbar puncture (n=86). Fecal microbiome composition was characterized using 16S rRNA sequencing. A QIIME2/Phyloseq pipeline was used to complete denoising, feature classification, filtration of rare taxa and rarefaction to even sampling depth. Cerebrospinal fluid (CSF) biomarkers were quantified using the NeuroToolKit panel of robust prototype assays (Roche Diagnostics International Ltd, Rotkreuz, Switzerland). Multiple regression tested for associations between alcohol consumption and global cognitive function indicated by Preclinical Alzheimer’s Cognitive Composite (PACC3) scores (Donahue et al, 2014; Jonaitis et al, 2019), in addition to associations with CSF biomarkers. Non‐parametric methods tested associations between alcohol consumption and phylogenetic diversity as well as bacterial community structure.

**Result:**

A nonsignificant quadratic trend was observed between alcohol consumption and PACC3 scores (Figure 1). No significant associations were observed between alcohol and CSF biomarkers (Table 2). When testing associations between alcohol and gut microbiome independent from CSF and cognitive outcomes, no significant association was observed between alcohol consumption and phylogenetic diversity, nor was alcohol associated with significant alterations to bacterial community structure.

**Conclusion:**

Associations between alcohol consumption and cognition as well as CSF biomarkers were expected to be partially mediated by gut microbial alterations, but few relationships were observed, apart from a nonlinear trend between alcohol and PACC3. Of note, few individuals reported heavy alcohol use, which may have limited power to detect effects in this sample.